# Dr. Stewart Lockie

**Published:** 1910-05-07

**Authors:** 


The death is announced, at Stanwix, near Carlisle, of Dr. Stewart Lockie, in his seventy-fifth year. He was educated at Kelso Grammar School and Edinburgh University. In 1858, after becoming a licentiate of the Royal College of Surgeons, he became an M.D. of his University and was appointed house surgeon to the Carlisle Dispensary, where for a time he taught reading and arithmetic to the working men of the town. In 1861 he began in private practice. He was appointed honorary physician in 1868 to the Cumberland Infirmary, where he remained for thirty years. He was a president of the Cumberland and Westmorland branch of the British Medical Association in 1877.

